# Activation of GLP-1R ameliorates alcohol withdrawal induced anxiety-like behavior by regulating neuronal mitochondrial quality control

**DOI:** 10.3389/fphar.2026.1820128

**Published:** 2026-05-29

**Authors:** Ziqi Wang, Wei Zhao, Xiaofei Chen, Shuang Zhao, Xiaotong Li, Quanwei Yang, Fangjiao Zong, Hanting Zhang

**Affiliations:** 1 Department of Pharmacology, Qingdao University School of Pharmacy, Qingdao, China; 2 Kedou Brain-Computer Technology Co., Ltd., Suzhou, China; 3 Shandong Key Laboratory of Pathogenesis and Prevention of Brain Diseases, Qingdao, China; 4 School of Pharmacy, Jiangxi Medical College, Nanchang University, Nanchang, China

**Keywords:** alcohol withdrawal, anxiety, CREB, GLP-1r, mitochondrial function

## Abstract

**Introduction:**

Alcohol use disorder (AUD) is a specific psychological state induced by repeated heavy drinking, and withdrawal symptoms such as anxiety are closely related to relapse after withdrawal. While neuronal damage caused by alcohol is considered a significant precipitating factor for withdrawal-induced anxiety, the underlying molecular mechanisms remain unclear.

**Methods:**

In this study, we established a mouse model of alcohol withdrawal through 3 months of chronic ethanol exposure (CEE) followed by withdrawal. Mice were treated with semaglutide (0.03 mg/kg) via intraperitoneal injection and subjected to behavioral, biochemical, and morphological analyses.

**Results:**

Our results demonstrate that the glucagon-like peptide-1 receptor (GLP-1R) agonist semaglutide alleviates anxiety-like behaviors in CEE withdrawal mice and reverses the downregulation of GLP-1R and its downstream effector CREB in the mitochondria of prefrontal cortex (PFC) neurons. Enhancing the GLP-1R/CREB pathway regulates mitochondrial quality control, including fission, fusion, and mitophagy, to maintain mitochondrial function and ameliorate synaptic impairment.

**Discussion:**

These findings suggest that activation of GLP-1R ameliorates alcohol withdrawal-induced anxiety-like behaviors by regulating neuronal mitochondrial function, providing a potential therapeutic target for AUD.

## 1 Introduction

Alcohol use disorder (AUD) is a chronic relapsing disorder defined by a problematic pattern of alcohol use leading to clinically significant impairment or distress, as manifested by criteria such as compulsive alcohol use, loss of control over intake, continued drinking despite adverse consequences, and withdrawal symptoms (American Psychiatric Association, 2013). Withdrawal symptoms such as anxiety during withdrawal are closely linked to a high risk of relapses (Novak et al., 2003; Litman et al., 1979; Walter et al., 2006). Alcohol-induced neuronal damage, particularly synaptic dysfunction, may be a significant precipitating factor in alcohol withdrawal-induced anxiety; however, the specific molecular biological mechanism remains unclear. The prefrontal cortex (PFC) is a critical brain region regulating emotional processing (such as anxiety, fear), executive functions (such as decision-making, impulse control, and physiological responses to stress (Zong et al., 2023; Bridi et al., 2020), and it’s susceptible to chronic ethanol exposure and effective in regulating the anxiety symptoms caused by alcohol withdrawal in AUD (Patel, et al., 2022; George et al., 2012; Zahr and Pfefferbaum, 2017; Li W. et al., 2024). Human fMRI studies consistently show reduced gray matter volume and abnormal resting-state functional connectivity in the PFC of individuals with alcohol dependence (Daviet et al., 2022), with frontal regions being especially implicated in these effects (Makris et al., 2008; Rando et al., 2011; Zahr et al., 2017). This human evidence strongly suggests that investigating the molecular mechanisms of the PFC in animal models is an effective approach for translating clinical observations into mechanistic understanding—specifically, by precisely targeting the key nodes identified in human imaging studies to deconstruct the underlying therapeutic mechanisms at the molecular biology level. Converging evidence indicates that the PFC serves not only as a key hub for “top-down” emotion regulation within anxiety circuitry—exerting inhibitory control over limbic structures such as the amygdala (Kenwood et al., 2022; Myers-Schulz and Koenigns, 2012)—but also as a critical node for impulse control and decision-making in addictive behaviors (Friedman and Robbins, 2022; Jeong et al., 2026). In the chronic ethanol exposure (CEE) model, the PFC is involved in the regulation of anxiety induced by alcohol withdrawal or prolonged alcohol exposure (Pleil et al., 2015; Varodayan et al., 2018). Given that the Chronic Ethanol Exposure (CEE) paradigm captures features of both anxiety and addiction, targeting the PFC in this model offers a mechanistically specific approach to understanding how these two processes interact.

GLP-1R, a member of the G Protein-Coupled Receptor (GPCR) family primarily located on the plasma membrane, specifically binds to GLP-1 and plays a key role in regulating blood glucose levels and lipid metabolism (Zheng et al., 2024). Beyond its peripheral actions, GLP-1R also plays a key role in modulating energy homeostasis, including the central nervous system (CNS) (Barrera et al., 2011). In the CNS, GLP-1R is widely expressed in regions involved in reward, emotion, and cognition, such as the PFC, amygdala, hippocampus, and nucleus accumbens (Muscogiuri et al., 2017; Graham et al., 2020). In the PFC specifically, GLP-1R activation modulates synaptic transmission and dendritic plasticity (Csajbók et al., 2019; Yoon et al., 2020). Accumulating preclinical evidence has demonstrated broad neuroprotective effect of GLP-1R agonists in rodent models (Yun et al., 2018), with promising therapeutic outcomes recently reported in clinical trials for Parkinson’s disease (Athauda et al., 2017; Aviles-Olmos et al., 2013) and Alzheimer’s disease (Gejl et al., 2016). Detection in both animal and human samples revealed that chronic ethanol exposure significantly reduced GLP-1R levels in PFC (Torregrosa et al., 2026). Currently, clinical studies on GLP-1R for the treatment of alcohol use disorder have shown that GLP-1R agonists are associated with fewer alcohol-related medical events and significantly reduce alcohol use (Lähteenvuo et al., 2025). However, research on the molecular mechanisms by which GLP-1R agonists alleviate alcohol withdrawal-induced anxiety-like behaviors remains limited (Subhani M et al., 2024). Notably, a recent revealed that the GLP-1R agonist liraglutide modulates the mitochondrial quality control system in a mouse model of Parkinson’s disease, suggesting an important regulatory role of GLP-1R in central nervous system mitochondria (Wu P. et al., 2022).

Mitochondria are essential in regulating energy balance and are crucial for the cell’s energy production and maintain their quantity, structure, and function through a dynamic quality control system, including biogenesis, mitophagy, fusion, and fission (Pickles et al., 2018; Kiriyama and Nochi, 2017). Mitochondria play a crucial role in CNS (Tripathi and Ben-Shachar, 2024). In the CNS, mitochondria maintain neuronal function through oxidative phosphorylation (OXPHOS) (Deshwal et al., 2020; Pickles et al., 2018). OXPHOS in mitochondria is essential for maintaining neuronal function (Mattson MP et al., 2008). OXPHOS involves the coordinated action of five complexes (Complexes I–V: NDUFB8, SDHB, UQCRC2, MTCO1, ATP5A) in the synthesis of ATP. If one complex operates independently of the others, it will lead to an overall functional imbalance, thereby affecting energy production and compromising the normal basic functions of neuronal mitochondria (Sabini et al., 2023). Compelling pathological and genetic data defines mitochondria failure as the cause of numerous acquired nervous system diseases (Zong et al., 2024). Mitochondrial quality control is mediated by mitochondrial dynamic processes, coupled with continuous cycles of fission and fusion (Li Y.et al., 2024; Haileselassie et al., 2019; Song et al., 2024; Quintana-Cabrera and Scorrano, 2023). The specific molecular mechanism is as follows: mitochondrial fission is regulated by Dynamin-related protein 1 (DRP1), and its phosphorylation at serine 616 promotes fission. DRP1 interacts with FIS1 protein to participate in mitochondrial fission. Mitochondrial fusion is mediated by MFN1 and MFN2. Damaged mitochondria are eliminated through mitophagy. During this process, Pink1 accumulates and stabilizes on the outer mitochondrial membrane following mitochondrial damage; Pink1 and Parkin serve as key markers of mitophagy initiation. Subsequently, Pink1 activates the E3 ubiquitin ligase Parkin, thereby initiating a cascade of polyubiquitination targeting mitochondrial substrates. This ubiquitination modification facilitates the recognition of damaged mitochondria by the selective autophagy receptor P62; P62 acts as an autophagy receptor that links ubiquitinated cargo to the autophagosome, and its expression level can reflect autophagic degradation. P62 directly binds to LC3 protein through its LC3-interacting region and recruit mitochondria to the autophagic pathway (Radosavljevic et al., 2024). Meanwhile, cytosolic LC3-I undergoes lipidation to form LC3-II, which becomes embedded in the membrane of the expanding phagophore (Mizushima and Yoshimori, 2007). The LC3-II/LC3-I ratio is a marker of autophagy. In AUD, mitochondrial quality control is disrupted, manifesting as imbalanced mitochondrial dynamics (enhanced fission via DRP1 activation and reduced fusion via MFN1/2 downregulation), impaired mitophagy initiation (altered Pink1/Parkin signaling), and dysregulated autophagy (accumulation of P62 and abnormal LC3-II/I ratio) (French et al., 1972; Shang et al., 2020). Enhancing mitochondrial quality may therefore ameliorate neuronal damage and contribute to AUD treatment (Manzo-Avalos and Saavedra-Molina et al., 2010). However, whether GLP-1R signaling in the PFC can restore mitochondrial function thereby ameliorate alcohol withdrawal-induced anxiety remains unclear.

CREB, a ubiquitously expressed transcription factor, plays a pivotal role in neuroprotection. In neurons, it localizes to the mitochondrial matrix and directly binds to cAMP response elements (CRE) in the mitochondrial genome, indicating its crucial function in maintaining normal mitochondrial activity (Lee et al., 2005; Raefsky and Mattson, 2017; Li Z. et al., 2024). Alcohol exposure reduces CREB phosphorylation and activity in the PFC, contributing to neuroadaptations associated with dependence and withdrawal (Mons and Beracochea, 2016). Activation of GLP-1R promotes G protein-mediated cAMP production, which in turn triggers downstream PKA/CREB signaling to regulate cellular homeostasis (Xie et al., 2021; Gupta et al., 2025). Nevertheless, whether GLP-1R regulates mitochondria function through CREB remains unclear. Considering the essential roles of both GLP-1R and mitochondria in cellular energy metabolism, we hypothesize that GLP-1R/CREB signaling modulates neuronal mitochondrial quality control to preserve mitochondrial function, thereby influencing anxiety-like behaviors during alcohol withdrawal.

In addition to mitochondrial dysfunction, chronic alcohol exposure is known to impair neuronal damage (particularly synaptic dysfunction) in PFC. Postsynaptic density protein 95 (PSD95) is a critical scaffolding protein at excitatory synapses that organizes glutamate receptors and signaling complexes (Sturgill et al., 2009), while Synaptophysin (SYN) is a presynaptic vesicle protein essential for neurotransmitter release (Preobraschenski et al., 2025). Both markers are widely used to assess synaptic density and function (Glantz et al., 2007).

This study aims to investigate whether and how GLP-1R signaling in the PFC is involved in alcohol withdrawal-induced anxiety-like behavior, with a focus on mitochondrial quality control as a potential molecular mechanism. We propose the following hypothesis: chronic ethanol exposure downregulates the expression of GLP-1R in PFC neurons, leading to mitochondrial dysfunction via disruption of the CREB pathway. This impairment subsequently compromises neuronal synaptic function and ultimately contributes to the development of anxiety-like behavior. To test this hypothesis, we employ a chronic ethanol exposure (CEE) model and assess anxiety-like behavior using behavioral tests. We will then examine whether activation of GLP-1R with the agonist Semaglutide reverses the CEE-induced alterations at the molecular, mitochondrial, synaptic, and behavioral levels. Furthermore, we aim to determine whether the effects of GLP-1R activation on mitochondrial quality control and anxiety-like behavior are mediated through the CREB pathway. By integrating molecular biology, pharmacological, and behavioral tests, this study seeks to elucidate the role of a previously unrecognized mechanism—the regulation of mitochondrial function by the GLP-1R/CREB pathway in PFC neurons—in anxiety induced by CEE, thereby identifying a potential new therapeutic target for AUD.

## 2 Materials and Methods

### 2.1 Animals

Male C57BL/6J mice were obtained from Beijing Vital River Laboratory Animal Technology Co., Ltd. (Beijing, China). The mice were approximately 6–8 weeks old and weighed 20–25 g at the start of the experiment. Animals were housed (4–6 per cage) in plastic cages on a 12-h light/dark cycle (lights on at 8 a.m.) in a room with controlled temperature (24 °C ± 2 °C and relative humidity (40%–50%). Mice had free access to food and water. Animal procedures were approved by the Committee of Animal Experimental Ethics of Qingdao University, China. All efforts were made to minimize the number of animals used and their suffering.

### 2.2 Chronic ethanol exposure

The experimental groups were subjected to CEE for 90 days (Wang et al., 2020). Throughout the entire experimental period, food was available *ad libitum*. Ethanol was administered using a single-bottle paradigm, in which the sole fluid source provided was an ethanol solution at varying concentrations. In this paradigm, all fluid intake in the experimental group of mice was derived from the ethanol solution.

The ethanol concentration gradually increased to promote adaptation. Specifically, mice were first exposed to 10% ethanol (v/v) as the sole fluid source for 2 days, followed by 15% ethanol for 5 days. From day 8 onward, the ethanol concentration was maintained at 20% (v/v) for the remaining 83 days of the exposure period. The ethanol bottles were replaced with freshly prepared solutions once daily to ensure concentration stability and prevent contamination.

### 2.3 Experimental design

**FIGURE 1:**
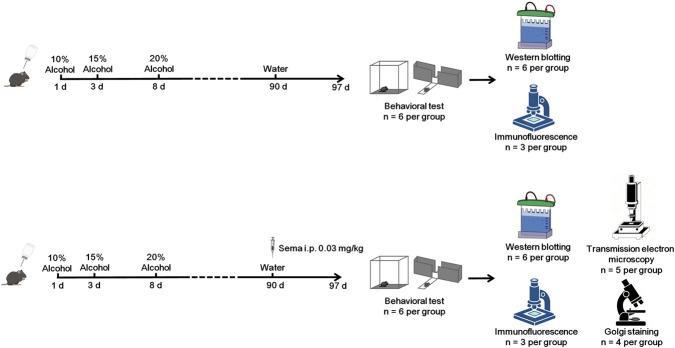
Schematic overview of the experimental protocol and animal allocation.

After obtaining the animals from Beijing Vital River Laboratory Animal Technology Co., Ltd., they acclimated to the shelter for 7 days. Constructing the ethanol withdrawal model: 18 mice were randomly divided into CEE and Water groups. After 90 consecutive days of CEE procedure, mice underwent a 7-day ethanol withdrawal period. After ethanol withdrawal period, all mice were subjected to behavioral tests, including the open field test (OFT) and the elevated plus maze (EPM). After the behavioral tests, mice were euthanized and brain tissues were collected for subsequent experiments, including Western blotting (n = 6 per group), immunofluorescence (n = 3 per group) (Figure 1).

To investigate the effects of the GLP-1R agonist semaglutide, fifty-seven mice were randomly assigned to three groups: Water, CEE, and CEE + Semaglutide groups. After 90 consecutive days of CEE procedure, mice underwent a 7-day ethanol withdrawal period. During the withdrawal period, mice in the CEE + Semaglutide group received daily intraperitoneal injections of semaglutide (0.03 mg/kg, HY-114118, MedChemExpress, United States of America) at 9:00 a.m. Water and CEE mice received intraperitoneal injections of the vehicle solution (1% DMSO +99% saline) saline vehicle at the same volume and schedule. The dose (0.03 mg/kg) selection was based on a thorough review of the literature. Specifically, previous studies investigating the effects of Semaglutide in rodent models of alcohol-related behaviors have reported effective doses of 0.026 mg/kg and 0.052 mg/kg (Aranäs et al., 2023), both of which significantly reduced alcohol-related behaviors. Given that both doses demonstrated efficacy, we selected an intermediate dose of 0.03 mg/kg to achieve a balance between efficacy and potential off-target effects. Twenty-four hours after the final injection, all mice were subjected to behavioral tests, including OFT and EPM. After the behavioral tests, mice were euthanized and brain tissues were collected for subsequent experiments, including Western blotting (n = 6 per group), immunofluorescence (n = 4 per group), transmission electron microscopy (TEM; n = 5 per group), and Golgi staining (n = 4 per group) (Figure 1).

### 2.4 Blood alcohol concentration (BAC) measurement

At the end of the CEE, before the 7-day withdrawal, blood sample was collected via tail snip to determine BAC. According to the manufacturer’s instructions for the AM1 Alcohol Analyser (Analox, UK), the blood was centrifuged to separate the serum. BAC was subsequently measured in mg/dL using a customized ethanol detection assay (Supplementary Figure S1 C).

### 2.5 Behavioral tests

#### 2.5.1 Open field test (OFT)

OFT was performed as described. The OFT was conducted in a white rectangular open field apparatus (50 × 50 × 50 cm). Each mouse was placed in a corner at the start of the test and recorded for 10 min by a camera located above the box. We cleaned the device with 75% alcohol after each trial. The tracking information was processed with the VisuTrack (Xin Ruan, China). The time spent in the center zone and the total distance traveled were recorded. The central area of the open field was 25% of the total area.

#### 2.5.2 Elevated plus maze (EPM)

The EPM consists of a cross-shaped platform with two open arms (25 × 8 × 12 cm) and two closed arms (25 × 8 × 12 cm). At the beginning of the experiment, the mice were placed in the center of the cross platform and facing the closed arm, and the mice were allowed to explore for 5 min and recorded by a camera located above the box. The time spent in the open and closed arms and the number of entries to the open and closed arms were recorded (Draghmeh and Fuehrlein, 2025).

### 2.6 Mitochondrial isolation

Mitochondria were isolated from mouse PFC tissue using a tissue mitochondrial extraction kit (Beyotime, C3606) according to the manufacturer’s instructions. The mitochondrial and cytosolic fractions were separated by differential centrifugation, resuspended in storage buffer containing phenylmethylsulphonyl fluoride. For Western blotting analysis, protein samples were mixed with 4x loading buffer, heated at 100 °C for 5–10 min to denature proteins, and then stored at −20 °C until use. Western blotting was used to confirm the successful separation of mitochondrial and cytosolic fractions, thereby validating the extraction procedure for subsequent studies (Supplementary Figure S1A). COXIV (Cytochrome c oxidase subunit IV) was used as a loading control for mitochondrial fractions, given its constitutive expression and enrichment in mitochondria. β-actin was used as a loading control for cytosolic fractions due to its predominant cytoplasmic localization.

### 2.7 Western blotting

PFC dissection was performed on ice under a stereomicroscope. The PFC was isolated based on anatomical landmarks: coronal sections from +2.0 mm to +1.0 mm relative to bregma (Paxinos and Franklin, 2001), with the medial-lateral boundaries defined as the medial prefrontal cortex (including the prelimbic and infralimbic cortices), and the ventral boundary set at the corpus callosum.

Primary antibodies against the following proteins were used: GLP-1R (ab218532, 1:1000, Abcam), CREB (ab32515, 1:1000, Abcam), OXPHOS (ab110413, 1:250, Abcam, Cambridge, UK), FIS1 (66635-1-IG, 1:2000, Proteintech), DRP1 (GB115659-50, 1:500, Servicebio), p-DRP1 (Ser616; ab314755, 1:1000, Abcam), MFN1 (66776-1-IG, 1:5000, Proteintech), MFN2 (67487-1-IG, 1:20000, Proteintech), LC3B (ab192890, 1:2000, Abcam), P62 (ab109012, 1:5000, Abcam), Pink1 (23274-1-AP, 1:500, Proteintech), Parkin (14060-1-AP, 1:2000, Proteintech), PSD95 (20665-1-AP, 1:1000, Proteintech), SYN (36406S, 1:1000, Cell Signaling Technology), COXIV (4D11-B3-E8, 1:1000, Cell Signaling Technology) and β-actin (81115–1-RR, 1:1000, Proteintech). The horseradish peroxidase-conjugated secondary tibodies were goat anti-mouse (ZB-2305, 1:3000, Zhongshan Golden Bridge) and goat anti-rabbit (ZB-2301, 1:3000, Zhongshan Golden Bridge). Detailed procedures for protein extraction, electrophoresis, transfer, and immunodetection are provided in the Supplementary Material (Supplementary Methods).

### 2.8 Correlation analysis

We performed individual-level correlation analyses using animals from both experimental cohorts (Water, n = 12; CEE, n = 12; CEE + Sema, n = 6). GLP-1R protein expression was quantified by Western blot, and anxiety-like behavior was assessed by OFT (Entries into the center area; Time in the center area (s)) and elevated plus maze EPM (Open-arm entries (%); Open-arm time (%)).

### 2.9 Immunofluorescence

Mice were perfused, and brain sections (30 μm) were prepared. Immunofluorescence staining was performed using primary antibodies against GLP-1R (GB113881, 1:5000, Servicebio) and NeuN (GB11138, 1:5000, Servicebio). Tyramide signal amplification (TSA) was used for signal enhancement according to the manufacturer’s instructions. Detailed procedures for perfusion, sectioning, blocking, antibody incubation, TSA staining, antibody stripping, and imaging are provided in the Supplementary Material. Images were acquired using a confocal microscope (Leica) and analyzed using ImageJ for quantification.

For quantification of GLP-1R-positive neurons, sections were coded and analyzed by an investigator blinded to group assignment. For each animal, three coronal sections spanning the PFC (bregma +1.69 mm) were selected. From each section, three non-overlapping fields of view were randomly selected from the mPFC under a ×20 objective. Only neurons with clear NeuN co-localization and a visible DAPI-stained nucleus were counted; neurons at the field edges were excluded. Automated cell counting was performed using ImageJ with a consistent threshold applied across all images. The threshold was established based on control sections and applied uniformly to all experimental groups.

For mean fluorescence intensity analysis, random regions were delineated within the medial prefrontal cortex. The mean fluorescence intensity of GLP-1R signal was measured using ImageJ after background subtraction. MFI values from each section were averaged per animal and normalized to the control group.

### 2.10 Transmission electron microscopy

Mice were anesthetized with isoflurane and then perfused with 40 ml of PBS (pH 7.4) followed by 40 ml of 2.5% (w/v) glutaraldehyde in 0.2 M phosphate buffer (pH 7.4). Brain tissues were quickly removed and fixed with the 2.5% (w/v) glutaraldehyde for 24 h with 4 °C. Brain tissues were rapidly removed, PFC tissues were isolated, followed by fixation with 2.5% (w/v) glutaraldehyde for 24 h at 4 °C. The sections were fixed in 1% osmium in the dark at room temperature for 2 h and then washed with distilled water. Then the tissues were successively immersed in 30%–50%-70%-80%-95%–100% alcohol for 20 min each time and 100% acetone twice for 15 min each time. The tissues were embedded and placed at 37 °C overnight followed by hardening at 60 °C for 48 h. The resin blocks were sectioned at 50 nm and placed on 150 mesh quadrate membrane copper mesh. The copper mesh was stained in 2% uranyl acetate saturated alcohol solution in the dark for 8 min. Wash with 70% alcohol; Wash with distilled water; 2.6% lead citrate solution was used to avoid carbon dioxide staining for 8 min. They were washed three times with distilled water. The copper mesh sections were dried overnight at room temperature in the copper mesh box. The images were collected under transmission electron microscope. For quantification of mitochondrial damage, at least 3 randomly selected fields per animal were analyzed (n = 5 mice per group). Mitochondrial damage was defined based on previously established criteria (Wu et al., 2014) as the presence of mitochondrial swelling (loss of normal ellipsoid shape) and/or cristae disruption (fragmentation, disorganization, or loss of cristae). The percentage of damaged mitochondria (% damage) was calculated as (number of mitochondria exhibiting swelling and/or cristae abnormalities/total number of mitochondria counted)) × 100%.

### 2.11 Golgi staining and sholl analysis

Golgi staining was conducted utilizing the Golgi staining kit (Catalog No. G1152, Servicebio Technology, Wuhan, China). The mice were placed in a gas anesthesia chamber containing isoflurane and anesthetized until the absence of pain reflexes, followed by decapitation. The excised brain was rinsed with double-distilled water, subsequently immersed in a pre-prepared staining solution (comprising equal proportions of liquid A and liquid B), stored in the dark at room temperature for 14 days. Thereafter, it was transferred to solution C for 7 days before being sectioned into 100-micron coronal slices using an oscillating microslicer. The sections were mounted on gelatin incubated in a mixture of D and E for 10 min. Following cleansing with double-distilled water, dehydration was performed using a series of ethanol concentrations, followed by three xylene washes prior to cover glass application. Subsequently, the Fiji software was used to trace and analyze the dendrites of pyramidal neurons in the PFC region.

For morphological analysis, five neurons per animal were selected from the PFC based on the following criteria: (1) complete impregnation with clearly visible dendrites; (2) isolated from neighboring neurons to avoid overlapping processes; (3) intact cell body and dendritic arbor within section. A total of 20 neurons were analyzed per experimental group (5 neurons × 4 mice). All image acquisition and quantification were performed by an investigator blinded to group assignment.

Neuronal images were captured under a ×40 objective using a light microscope (Leica). Sholl analysis was performed using ImageJ with the Sholl Analysis plugin. Concentric circles were drawn at 10-μm intervals from the soma, and the number of dendritic intersections at each distance was recorded. For statistical analysis, the mean number of intersections per distance per mouse was first calculated, and repeated-measures two-way ANOVA was performed with drug treatment as the between-subjects’ factor and distance from soma as the within-subjects’ factor (Zhao et al., 2025).

### 2.12 Statistical analysis

All experiments were analyzed by researchers blinded to the treatment conditions. All statistical analyses were conducted with the animal as the independent unit. Mice were randomly grouped and the sample sizes are indicated by dots in the figures or are stated in the figure legends. Normality was assessed using the Shapiro–Wilk test, and homogeneity of variances was assessed using Brown-Forsythe test. For comparisons between two groups, unpaired tests were used when assumptions of normality and homoscedasticity were met. For three independent groups, one-way ANOVA or two-way ANOVA was used, followed by Tukey *post hoc* test. When normality or homoscedasticity was violated, the Kruskal–Wallis test was performed, followed by Dunn’s *post hoc* test. All results were presented as mean ± standard errors of the mean (SEM), and P < 0.05 were considered statistically significant for all data.

## 3 Results

### 3.1 CEE induces anxiety-like behaviors and downregulates GLP-1R expression in mice

**FIGURE 2:**
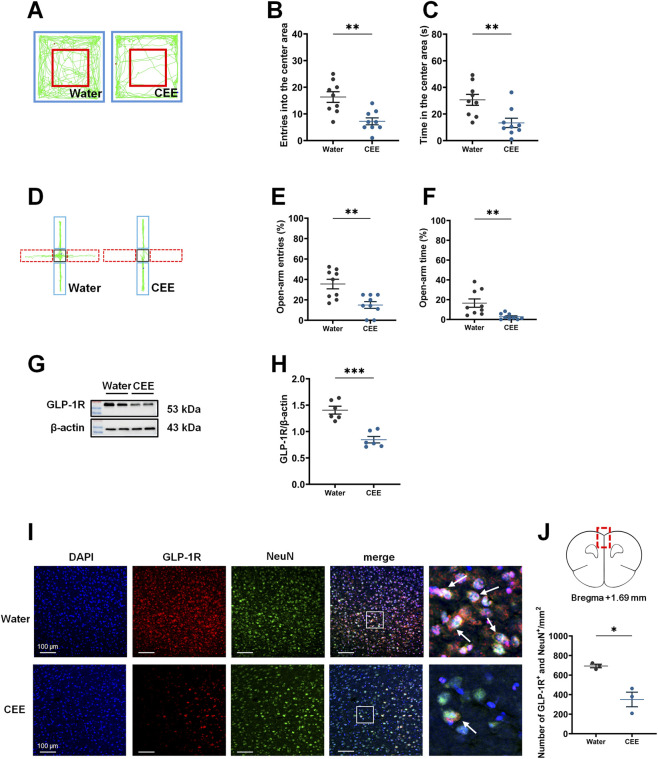
Effects of CEE on anxiety-like behaviors and GLP-1R expression in mice. **(A)** Representative tracking plot from the OFT. **(B)** Number of entries in the center area (**p = 0.0015. n = 9 mice per group). **(C)** Time spent in the center area (**p = 0.0056. n = 9 mice per group). **(D)** Representative track plot of the EPM test. **(E)** Number of entries in the open arms (**p = 0.0070. n = 9 mice per group). **(F)** Time spent in the open arms during the EPM test (**p = 0.0056. n = 9 mice per group). **(G,H)** Protein expression of GLP-1R was detected by Western blot analysis (***p = 0.0002. n = 6 mice per group). **(I)** Representative images of Immunofluorescence staining of GLP-1R and NeuN with DAPI nuclear counterstaining in the PFC. *p = 0.0113. n = 3 mice per group). NeuN (green) was used to label mature neurons, and DAPI (blue) was used as a nuclear counterstain. GLP-1R (red) was predominantly co-localized with NeuN-positive neurons, indicating neuronal expression. Scale bar = 100 μm. **(J)** PFC dissection: bregma +1.69 mm. All data are presented as mean ± standard error of the mean (SEM). *p < 0.05, **p < 0.01, ***p < 0.001. Abbreviations: CEE, Chronic ethanol exposure; GLP-1R, Glucagon-like Peptide-1 receptor; OFT, Open Field Test; EPM, Elevated Plus Maze.

We conducted the OFT and EPM test with Water mice and CEE mice. In the OFT (Figure 2A), CEE mice exhibited significantly reduced the entries (Figure 2B: Two-tailed unpaired t-test, t_(16)_ = 3.830, P < 0.01) and time (Figure 2C: Two-tailed unpaired t-test, t(16) = 3.199, P < 0.01) in the center area compared to water group mice. In the EPM (Figure 2D), CEE mice showed significantly decreased percentages of both entries (Figure 2E: Two-tailed unpaired t-test, t_(16)_ = 3.089, P < 0.01) and time (Figure 2F: Two-tailed unpaired t-test, t_(16)_ = 3.199, P < 0.01) in the open arms compared to water group mice.

We collected mouse PFC tissue to determine changes in GLP-1R levels. Western blotting analysis and immunofluorescence results showed that GLP-1R expression was significantly decreased in the PFC of CEE mice compared to water group mice (Figure 2I: Two-tailed unpaired t-test, t_(10)_ = 5.783, P < 0.001; Figure 2J: Two-tailed unpaired t-test, t_(4)_ = 4.446, P < 0.05). GLP-1R expression was significantly positively correlated with Entries into the center area, Time in the center area (s), Open-arm entries (%) and Open-arm time (%) (Supplementary Figure S1A-D). Consistent with the cell counting results, mean fluorescence intensity analysis revealed a significant decrease in GLP-1R fluorescence intensity in the CEE mice compared with the Water mice (Supplementary Figure S1D: Two-tailed unpaired t-test, t_(14)_ = 3.299, P < 0.05). These findings indicate that CEE may induce anxiety-like behaviors and lead to a decrease in the expression level of GLP-1R specifically in neurons within the PFC.

### 3.2 GLP-1R agonist semaglutide treatment ameliorates CEE induced anxiety-like behaviors and reverses the downregulation of GLP-1R expression

**FIGURE 3:**
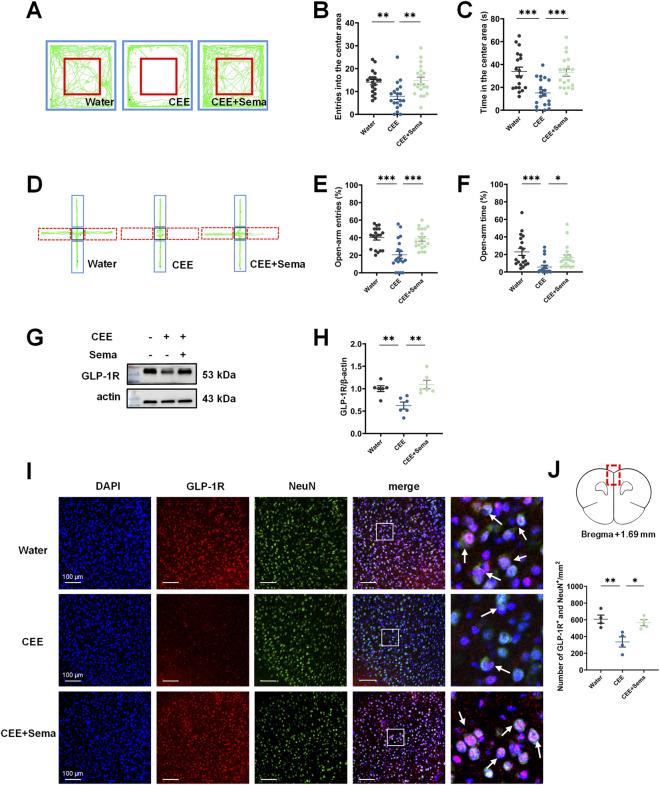
Effects of Semaglutide on CEE-induced anxiety-like behaviors and GLP-1R expression. **(A)** Representative tracking plot from the OFT. **(B)** Number of entries in the center area (**p < 0.01. n = 19 mice per group). **(C)** Time spent in the center area (***p < 0.001. n = 19 mice per group). **(D)** Representative track plot of the EPM test. **(E)** Number of entries in the open arms (***p < 0.001. n = 19 mice per group). **(F)** Time spent in the open arms during the EPM test (*p < 0.05, ***p < 0.001. n = 19 mice per group). **(G,H)** Protein expression of GLP-1R was detected by Western blot analysis (**p < 0.01. n = 6 mice per group). **(I)** Representative confocal images of Immunofluorescence staining of GLP-1R and NeuN with DAPI nuclear counterstaining in the PFC. (*p < 0.05, **p < 0.01. n = 4 mice per group). NeuN (green) was used to label mature neurons, and DAPI (blue) was used as a nuclear counterstain. GLP-1R (red) was predominantly co-localized with NeuN-positive neurons, indicating neuronal expression. Scale bar = 100 μm. **(J)** PFC dissection: bregma +1.69 mm. Data are presented as mean ± SEM. *p < 0.05, **p < 0.01, ***p < 0.001. Abbreviations: CEE, Chronic ethanol exposure; GLP-1R, Glucagon-like Peptide-1 receptor; OFT, Open Field Test; EPM, Elevated Plus Maze; Sema, Semaglutide.

Semaglutide was injected into CEE mice to detect whether activation of GLP-1R ameliorates anxiety-like behaviors. We used another batch of mice to form Water, CEE, and Semaglutide treatment group. In the OFT, Semaglutide treatment significantly improved the CEE-induced reduction in the number and time of entry into the center area (Figures 3A,B: one-way ANOVA, F_(2, 54)_ = 7.699, P = 0.0011; Tukey *post hoc* test, P < 0.01; Figure 3C: one-way ANOVA, F_(2, 54)_ = 10.42, P = 0.0001; Tukey *post hoc* test, P < 0.001). In the EPM test, Semaglutide also significantly reversed the reduction in the number and time of entry in the open arms of CEE mice (Figure 3E: one-way ANOVA, F_(2, 54)_ = 7.637, P = 0.0004; Tukey *post hoc* test, P < 0.05; Figure 3F: one-way ANOVA, H = 19.41, P < 0.001; Dunn’s *post hoc* test, P < 0.01, P < 0.001).

To further assess the effects of GLP-1R agonist Semaglutide on PFC brain region, the expression level of GLP-1R were evaluated. Western blotting analysis and immunofluorescence results showed that GLP-1R activation effectively reversed the downregulation of GLP-1R protein expression in prefrontal cortical neurons of CEE mice (Figure 3I: one-way ANOVA, F_(2, 15)_ = 9.666, P = 0.0020; Tukey *post hoc* test, P < 0.01; Figure 3J: one-way ANOVA, F_(2, 9)_ = 8.677, P = 0.0079; Tukey *post hoc* test, P < 0.05, P < 0.01). GLP-1R expression was significantly positively correlated with Entries into the center area, Time in the center area (s), Open-arm entries (%) and Open-arm time (%) (Supplementary Figure S2A-D). Consistent with the cell counting results, mean fluorescence intensity analysis revealed a significant decrease in GLP-1R fluorescence intensity in the CEE mice compared with Water mice, while semaglutide treatment significantly restored GLP-1R intensity (Supplementary Figure S1E: one-way ANOVA, F_(2, 9)_ = 6.495, P = 0.0180; Tukey *post hoc* test, P < 0.05). These results suggest that GLP-1R activation with Semaglutide ameliorates CEE-induced anxiety-like behaviors and restores GLP-1R expression specifically in PFC neurons.

### 3.3 GLP-1R agonist semaglutide ameliorates mitochondrial damage in CEE mice

**FIGURE 4:**
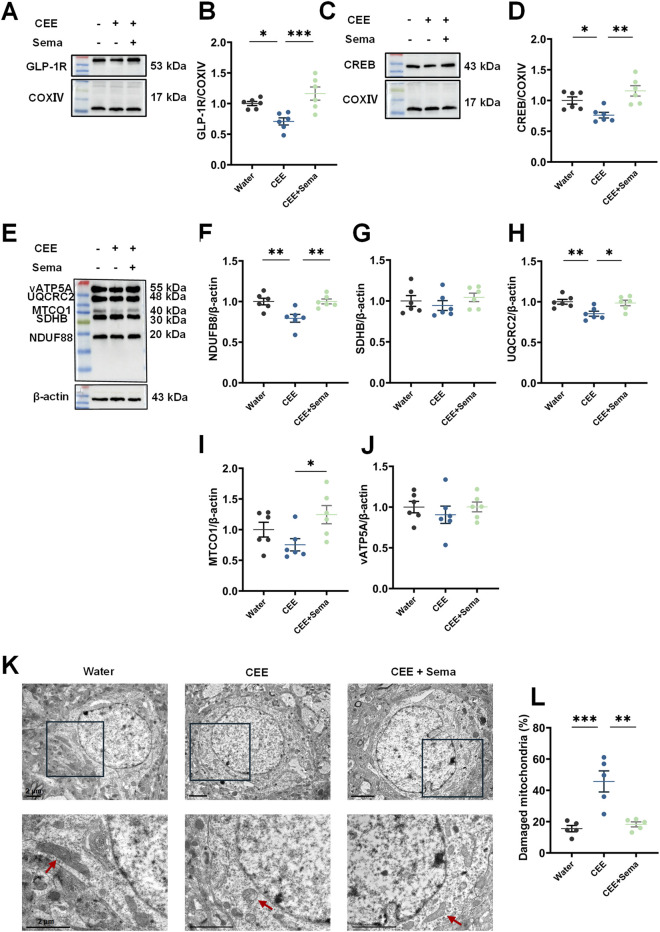
Effects of Semaglutide on mitochondrial damage in CEE mice. **(A,B)** Protein expression of mitochondrial GLP-1R was detected (*p < 0.05, ***p < 0.001. n = 6 mice per group). **(C,D)** Protein expression of mitochondrial CREB was detected by Western blot analysis (*p < 0.05, **p < 0.01. n = 6 mice per group). **(E-J)** Protein expression of mitochondrial OXPHOS was detected by Western blot analysis, including complex I (NDUFB8), complex II (SDHB), complex III (UQCRC2), complex IV (MTCO1), and complex V (ATP5A) (*p < 0.05, **p < 0.01. n = 6 mice per group). **(K)** Mitochondrial morphology of neurons in PFC of different groups. Scale bar = 2 μm. **(L)** Ratio of damaged mitochondria (Representative TEM images were used to calculate the percentage of damaged mitochondria. Mitochondrial damage was defined as the presence of mitochondrial swelling and/or cristae disruption, as described in the Materials and Methods.) (**p < 0.01, ***p < 0.001. n = 5 mice per group). Data are presented as mean ± SEM. *p < 0.05, **p < 0.01, ***p < 0.001. Abbreviations: GLP-1R, Glucagon-like Peptide-1 receptor; CREB, cAMP Response Element-Binding; OXPHOS, Oxidative phosphorylation; Sema, Semaglutide; NDUFB8, Ubiquinone oxidoreductase subunit B8; SDHB, Succinate dehydrogenase complex iron sulfur subunit B; UQCRC2, Ubiquinol-cytochrome c reductase core protein 2; MTCO1, Mitochondrially encoded cytochrome c oxidase I; vATP5A, ATP synthase F1 subunit alpha.

Changes in GLP-1R protein levels within mitochondria were detected by Western blot. The results showed that GLP-1R expression was significantly downregulated in the mitochondria of CEE mice, and this downregulation was reversed by treatment with Semaglutide (Figures 4A,B: one-way ANOVA, F_(2, 15)_ = 9.957, P = 0.0018; Tukey *post hoc* test, P < 0.05, P < 0.001). We subsequently assessed CREB expression in mitochondria. Western blotting analysis revealed a significant downregulation of CREB in CEE mice, which was also reversed following the administration of the GLP-1R agonist Semaglutide (Figures 4C,D: one-way ANOVA, F_(2, 15)_ = 9.383, P = 0.0023; Tukey *post hoc* test, P < 0.01).

We quantified the expression levels of OXPHOS proteins (complex I-V) to evaluate the mitochondrial function. Significant downregulation was observed in Mitochondrial Complex I NDUFB8 and Complex III UQCRC2. GLP-1R agonist Semaglutide significantly reversed these decreases (Figures 4F,H: one-way ANOVA, F_(2, 15)_ = 8.705, P = 0.0031, F_(2, 15)_ = 6.600, P = 0.0088; Tukey *post hoc* test, P < 0.05, P < 0.01). Mitochondrial Complex IV MTCO1 showed a downward trend, GLP-1R activation significantly reversed this trend (Figure 4I: one-way ANOVA, F_(2, 15)_ = 3.908, P = 0.0430; Tukey *post hoc* test, P < 0.05). Mitochondrial Complex V vATP5A and Complex II SDHB levels exhibited no significant changes (Figures 4G,J). We also examined mitochondrial morphology by using transmission electron microscope (TEM). Compared with the control group mice, CEE-exposed mice exhibited mitochondrial swelling accompanied by cristae disruption or loss, indicating mitochondrial damage. TEM analysis revealed that CEE exposure induced marked mitochondrial damage in PFC, characterized by mitochondrial swelling and/or cristae disruption. Quantification of the percentage of damaged mitochondria showed a significantly higher percentage of damaged mitochondria in the CEE mice compared with controls, while semaglutide treatment significantly attenuated this effect. (Figures 4K,L: one-way ANOVA, F_(2, 12)_ = 15.95, P = 0.0004; Tukey *post hoc* test, P < 0.01, P < 0.001). These results suggest that GLP-1R activation is associated with mitochondrial functional impairment, potentially involving the GLP-1R/CREB signaling pathway.

### 3.4 GLP-1R agonist semaglutide ameliorates mitochondrial function in CEE mice by regulating mitochondrial quality control

**FIGURE 5:**
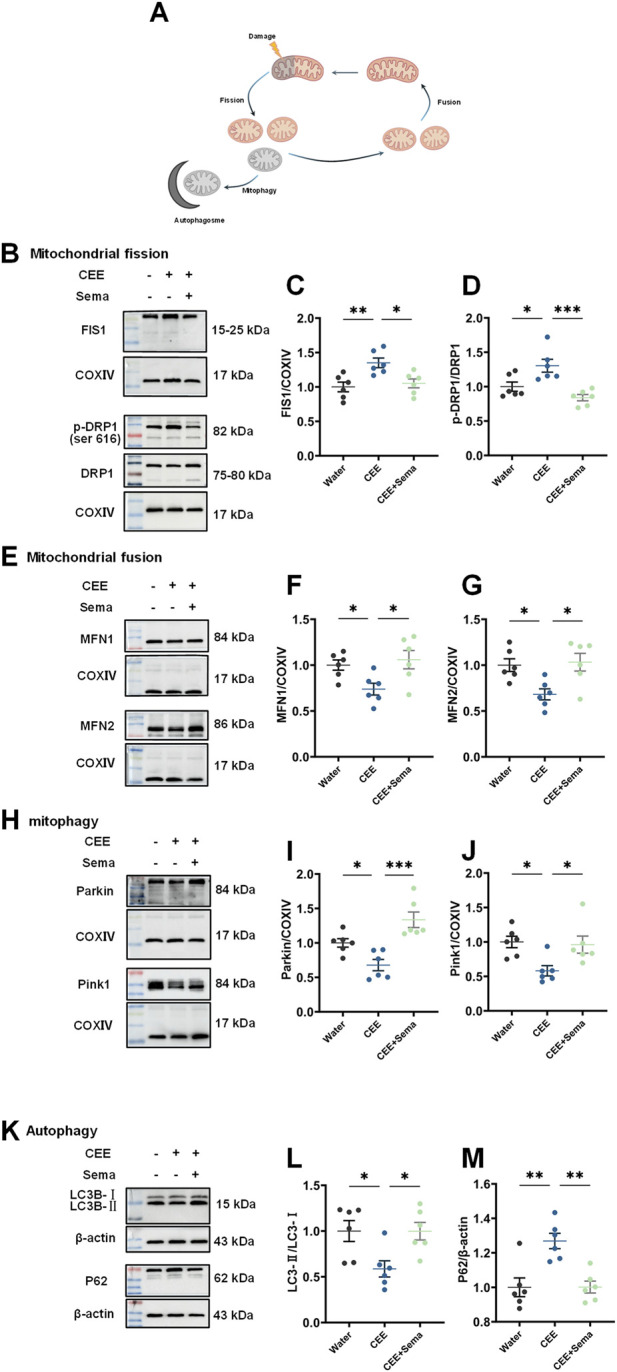
Regulation of mitochondrial quality control by Semaglutide in CEE mice. **(A)** Mitochondrial quality control, including fission, fusion, and mitophagy. **(B–D)** Protein expression of mitochondrial FIS1, DRP1 and p-DRP1 were detected (*p < 0.05, **p < 0.01, ***p < 0.001. n = 6 mice per group). **(E–G)** Protein expression of mitochondrial MFN1 and MFN2 were detected (*p < 0.05. n = 6 mice per group). **(H–J)** Protein expressions of mitochondrial Parkin and Pink1 were detected *p < 0.05, ***p < 0.001. n = 6 mice per group). **(K-M)** Protein expressions of LC3B and P62 were detected (*p < 0.05, **p < 0.01. n = 6 mice per group). Data are presented as mean ± SEM. *p < 0.05, **p < 0.01, ***p < 0.001. Abbreviations: GLP-1R, Glucagon-like Peptide-1 receptor; Sema, Semaglutide; DRP1DRP1, Dynamin-related protein-1; FIS1, Fission, mitochondrial 1; MFN1, Mitofusin 1; MFN2, Mitofusin 2; Pink1, PTEN-induced kinase 1; Parkin, Parkin RBR E3 ubiquitin-protein ligase; LC3, Microtubule-associated protein 1A/1B-light chain 3.

Mitochondrial quality control, including fission, fusion, and mitophagy, is critical for maintaining mitochondrial homeostasis (Figure 5A) (Virga et al., 2024). The expression of mitochondrial fission-related protein phospho-DRP1 (ser 616) and FIS1 were increased in CEE mice, suggesting an increase in mitochondrial fragmentation. Activation of the GLP-1R significantly downregulated the expression of these proteins and alleviated mitochondrial fragmentation (Figures 5B–D: one-way ANOVA, F_(2, 15)_ = 7.701, P = 0.0050, F_(2, 15)_ = 0.06364, P = 0.05429, F_(2, 15)_ = 10.90, P = 0.0012; Tukey *post hoc* test, P < 0.05, P < 0.01, P < 0.001). In addition, CEE mice exhibited significantly downregulated expression of mitochondrial fusion proteins MFN1 and MFN2 compared to water group mice. Semaglutide treatment effectively restored these deficits (Figures 5E–G: one-way ANOVA, F_(2, 15)_ = 6.600, P = 0.0088, F_(2, 15)_ = 6.360, P = 0.0100; Tukey *post hoc* test, P < 0.05).

Mitophagy: Pink1 stabilizes Parkin and recruits it to mitochondria to trigger mitophagy. CEE mice displayed marked mitophagy dysfunction, characterized by a significantly downregulated of Pink1 and Parkin (Figures 5H–J: one-way ANOVA, F_(2, 15)_ = 14.08, P = 0.004, F_(2, 15)_ = 5.751, P = 0.0140; Tukey *post hoc* test, P < 0.05, P < 0.001). Autophagy: The ratio of autophagy-related protein LC3-II/LC3-I was significantly downregulated and P62 accumulation was increased in CEE mice compared with water group mice, indicating impaired mitochondrial clearance (Figures 5K–M: one-way ANOVA, F_(2, 15)_ = 5.650, P = 0.0148, F_(2, 15)_ = 11.73, P = 0.0009; Tukey *post hoc* test, P < 0.05, P < 0.01). Semaglutide effectively rescued these abnormalities; normalizing markers associated with autophagy and mitophagy. Taken together, these results suggest that the GLP-1R agonist Semaglutide is associated with improvement of mitochondrial dysfunction in CEE mice, and this effect may involve regulation of mitochondrial quality control mechanisms, encompassing fission, fusion, mitophagy and autophagy.

### 3.5 GLP-1R agonist semaglutide ameliorates neuronal synaptic damage in the PFC of CEE mice

**FIGURE 6:**
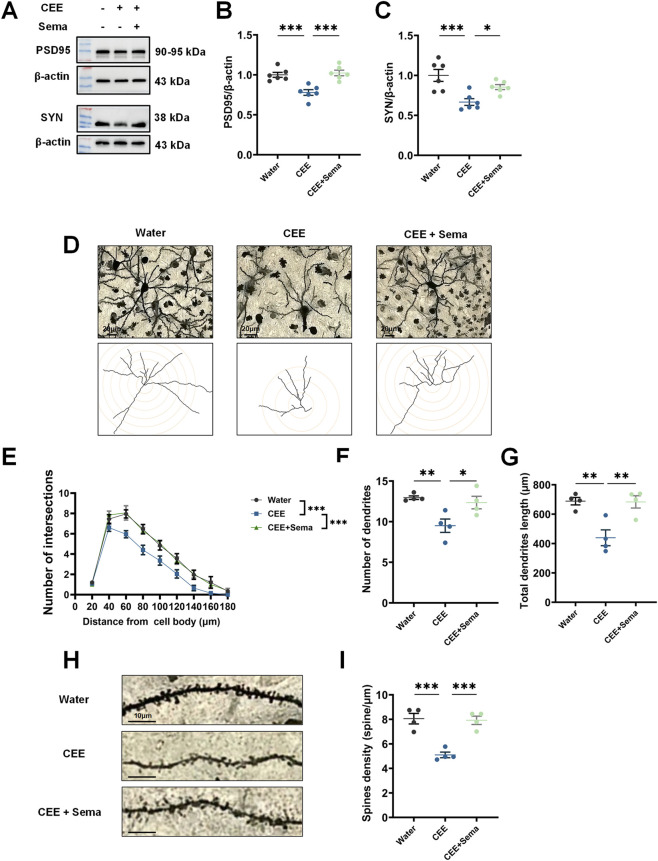
Synaptic morphology in PFC pyramidal neurons of CEE mice treated with Semaglutide. **(A-C)** Protein expressions of PSD95 and SYN were detected (*p < 0.05, ***p < 0.001. n = 6 mice per group). **(D)** Representative images of neurons in PFC labeled using Golgi staining. Scale bar = 20 μm. **(E)** Sholl analysis of PFC neurons revealing alterations in basal dendritic intersections at distinct distances from the soma. Two-way ANOVA with repeated measures revealed a significant effect of drug treatment and distance from the soma (*p < 0.05, **p < 0.01, ***p < 0.001. n = 4 mice per group). **(F,G)** Total dendritic length and dendritic branch number (**p < 0.01. n = 4 mice per group). **(H)** Representative images of dendritic spines by Golgi staining. **(I)** Spine density. Scale bar = 10 μm. (***P < 0.001. n = 4 mice per group). Data are presented as mean ± SEM. *p < 0.05, **p < 0.01, ***p < 0.001. Abbreviations: GLP-1R, Glucagon-like Peptide-1 receptor; Sema, Semaglutide; PSD95, Postsynaptic density protein 95; SYN, Synuclein.

Mitochondria quality control system is critical for maintaining neuronal function. We further assessed the expression of key synaptic structural proteins in neurons. Western blotting results showed that the expression levels of the synaptic proteins PSD95 and SYN were significantly reduced in CEE mice compared to water mice, and Semaglutide treatment effectively ameliorated these deficits (Figures 6A–C: one-way ANOVA, F_(2, 15)_ = 15.61, P = 0.0002, F_(2, 15)_ = 9.999, P = 0.0017; Tukey *post hoc* test, P < 0.05, P < 0.001).

We subsequently examined synaptic morphological structure. PFC was stained using the Golgi staining to visualize the complexity of dendrites. Representative images and reconstructed dendritic structures are shown in Figure 5D. Concentric circles spaced at 20 μm intervals were drawn, and the number of intersections between the concentric circles and dendrites was calculated as a parameter to evaluate the complexity of the dendritic tree. Sholl analysis showed that the number of dendritic intersections varied at different radial distances from the cell body. Notably, the number of dendritic intersections was significant decreased in CEE mice (Figure 6E: Two-way ANOVA, drug effect: F_(2, 9)_ = 30.37, P < 0.0001; distance effect: F_(14, 126)_ = 166.4, P < 0.0001; Tukey’s *post hoc* test, P < 0.05, P < 0.01, P < 0.001). Semaglutide significantly attenuated this reduction, leading to a restoration level comparable with controls. Semaglutide also effectively prevented the CEE-induced decreases in total dendritic length and total number of dendrites (Figure 6F: One-way ANOVA, F_(2, 9)_ = 7.673, P = 0.0114; Tukey *post hoc* test, P < 0.05; Figure 6G: One-way ANOVA, F_(2, 9)_ = 11.51, P = 0.0033; Tukey *post hoc* test, P < 0.01). Simultaneously, Semaglutide reversed the reduction in dendritic spine density (Figures 6H,I). These results demonstrated that GLP-1R activation ameliorates synaptic impairment in the prefrontal cortex of CEE mice.

## 4 Discussion

AUD is a chronic and highly prevalent mental disorder characterized by negative emotional states such as anxiety during alcohol withdrawal, which may follow a chronic relapsing course (Grant et al., 2015; Anker and Kushner, 2019). GLP-1R has traditionally been studied for its role in regulating blood glucose and energy metabolism. Recent studies have revealed its additional functions in modulating alcohol consumption and alcohol-induced cognitive decline (Aranäs et al., 2023; Liu et al., 2024). However, whether GLP-1R can regulate anxiety-like behaviors during alcohol withdrawal and the underlying biological mechanisms remain unclear and warrant further investigation. In this study, our findings suggest a role for GLP-1RA Semaglutide can alleviate alcohol withdrawal-induced anxiety-like behavior, potentially involving modulation of mitochondrial quality control (including fission, fusion, and mitophagy). This may offer a new theoretical basis for developing novel treatments for AUD.

In this study, we found that in a mouse model of anxiety-like behavior induced by CEE, which is consistent with previous findings (Zhao et al., 2024). The GLP-1R agonist semaglutide effectively alleviated anxiety-like behavior. GLP-1R specifically binds to its key ligand, the hormone GLP-1, and plays a central role in regulating blood glucose levels and lipid metabolism in humans (MacDonald et al., 2022; Bu et al., 2024). Recent research on GLP-1R in the CNS has demonstrated its neuroprotective effects, including improving cognition and memory, reducing neuroinflammation, among others (Siddeeque et al., 2024; Roy et al., 2025). In addition, studies on the role of GLP-1R in AUD have revealed that GLP-1R activation can alleviate behaviors such as anxiety and memory impairment (Liu et al., 2024). However, the underlying mechanism remains unclear. In our study, CEE induces anxiety-like behaviors in mice, which is consistent with previous findings (Zhao et al., 2024). GLP-1R agonist Semaglutide alleviated anxiety-like behaviors in CEE mice. Through further mechanistic exploration, we found that GLP-1R activation may alleviate alcohol withdrawal-induced anxiety by regulating mitochondrial function through mitochondrial quality control processes.

This study suggests that chronic ethanol exposure downregulated GLP-1R expression in neurons of the PFC, which is consistent with previous studies (Lähteenvuo et al., 2025). Treatment with the GLP-1R agonist semaglutide reversed this change, returning GLP-1R expression to near-normal levels. These findings suggest that CEE may induce anxiety-like behavior in mice by impairing GLP-1R expression in the PFC, and that semaglutide ameliorates these behaviors by upregulating GLP-1R expression. Previous studies have demonstrated that GLP-1R is distributed in several brain regions, including the PFC, amygdala, and hippocampus (Yang et al., 2022). Considering that PFC is a key region involved in emotional regulation (Suzuki and Tanaka, 2021) and is closely linked to the pathogenesis of alcohol-withdrawal-induced anxiety, we chose to focus on the PFC for our investigation.

Based on the observation that mitochondrial GLP-1R expression was decreased in CEE mice and reversed by semaglutide treatment, we formulated the following hypothesis. Early and recent studies indicate that ligand-receptor complexes are internalized via an orderly endocytic pathway (Goldstein et al., 1979; Marks et al., 1996; Bonifacino and Dell’Angelica, 1999; Bonifacino and Traub, 2003; Mellman, 1996; von Zastrow and Sorkin, 2007; Kirchhausen, 1999; Sorkin and Von Zastrow, 2002; Conner and Schmid, 2003), forming early endosomes (Pandey, 2009). A subset of GPCRs is transported to other organelles, such as mitochondria, the Golgi apparatus, or the endoplasmic reticulum, via specialized vesicles or membrane contact sites after endocytosis (Stäubert and Schoneberg, 2017; Fasciani et al., 2022; Rehman et al., 2025). Chronic ethanol exposure is known to disrupt endocytic trafficking and organelle communication (Gołaszewski et al., 2025), potentially altering the intracellular distribution of GPCRs including GLP-1R. By isolating mitochondrial and cytoplasmic fractions and performing Western blot analysis, we detected the presence of GLP-1R in mitochondria, with its expression level significantly differing from that in the cytoplasm (Supplementary Figure S1A, B). This suggests that GLP-1R may localize to the mitochondrial membrane system. Therefore, we hypothesize that GLP-1R agonists may be delivered to mitochondria via endocytosis, thereby influencing GLP-1R signaling pathways within mitochondria. We therefore hypothesize that in CEE mice, impaired endocytic trafficking may disrupt the delivery of GLP-1R to mitochondria, contributing to mitochondrial dysfunction and anxiety-like behavior; conversely, GLP-1R agonists such as semaglutide may restore this endocytic delivery, thereby activating mitochondrial GLP-1R signaling and exerting protective effects.

Further mechanistic investigation revealed that in CEE mice, the PFC exhibited not only downregulated GLP-1R expression but also decreased CREB expression. As a widely expressed transcription factor critical for the regulation of mitochondrial gene expression, CREB plays an important role in neuroprotection (Signorile et al., 2020). Based on these findings, we hypothesized that activation of GLP-1R could effectively alleviate CEE-induced mitochondrial damage in the prefrontal cortex. To test this hypothesis, we investigated mitochondrial OXPHOS and morphology. Our results showed that the expression of key components of mitochondrial oxidative phosphorylation complexes, UQCRC2 and NDUFB8, was significantly reduced, and these changes were reversed by treatment with the GLP-1R agonist semaglutide. TEM further revealed mitochondrial swelling and/or disruption of cristae structure in CEE mice, which were markedly improved following semaglutide treatment. As the core of cellular metabolism, alterations in mitochondrial OXPHOS can lead to mitochondrial dysfunction (Vercellino and Sazanov, 2022; Nicholls and FL, 2013). However, mitochondrial damage is primarily characterized by swelling and/or disruption of cristae structure (Wu et al., 2014), and the morphological observations in this study are consistent with these features. These findings suggest that the GLP-1R/CREB pathway may be associated with the regulation of mitochondrial OXPHOS, which may contribute to the preservation of normal mitochondrial function. Given that GLP-1R and mitochondria share common roles in the regulation of energy metabolism, this study first focused on alterations in the mitochondrial GLP-1R/CREB pathway.

GLP-1R has been shown to play an important role in mitochondrial function (Timper et al., 2020), and our findings link GLP-1R activation to the restoration of normal levels of mitochondrial fission and fusion. Mitochondria are highly dynamic organelles that continuously undergo fission and fusion to maintain a functional mitochondrial pool (Mishra and Chan, 2016). Mitochondrial fission is regulated by Dynamin-Related Protein 1 (DRP1), whose phosphorylation at Ser616 promotes fission (Wu L. K. et al., 2022). DRP1 interacts with FIS1 protein to facilitate mitochondrial fission (Kleele et al., 2021). Mitochondrial fusion is mediated by MFN1 and MFN2 (Chen et al., 2003). Under alcohol-induced conditions, mitochondrial quality control is disrupted, characterized primarily by excessive fission due to DRP1 upregulation, reduced fusion via downregulation of MFN1/2 (Liu et al., 2022). Similarly, we observed an imbalance in mitochondrial dynamics within the PFC of CEE mice. Specifically, mitochondrial fission was increased (as indicated by an elevated pDRP1/DRP1 ratio and upregulation of FIS1), while mitochondrial fusion was impaired (as evidenced by downregulation of MFN1/2). Consistent with our results, previous *in vivo* and *in vitro* studies have demonstrated that alcohol exposure leads to increased mitochondrial fission and reduced fusion (Abdallah and Singal, 2020; Siggins et al., 2023; Shang et al., 2020). The GLP-1R agonist semaglutide reversed these alterations. In this study, we hypothesize that GLP-1R also exerts a significant effect within mitochondria of PFC in CEE mice. Our findings suggest that GLP-1R activation is associated with normalized levels of mitochondrial fission and fusion markers.

Imbalance in mitochondrial dynamics is often accompanied by abnormal mitophagy and autophagy (Medala et al., 2021). Mitophagy is associated with various brain disorders (Picca et al., 2023; Fang et al., 2019) and plays an important role in alcohol-induced brain injury (Ruiter-Lopez et al., 2025). PINK1 stabilizes Parkin and recruits it to mitochondria to initiate mitophagy (Vives-Bauza et al.,2010). Both proteins are linked to numerous brain diseases (Imberechts et al., 2022; Mao et al., 2022). Meanwhile, the LC3-II/LC3-I ratio and P62 are well-established markers of autophagy (Liu et al., 2013). Under alcohol-induced conditions, mitophagy and autophagy are disrupted, characterized primarily by impaired initiation of mitophagy resulting from altered Pink1/Parkin signaling, and abnormal autophagy manifested by P62 accumulation and an aberrant LC3-II/I ratio (Thoudam et al., 2024). These findings are consistent with our results, which show that under the pathological state induced by CEE, both PINK1 and Parkin are decreased, suggesting that CEE may be associated with abnormal mitophagy; P62 is upregulated and the LC3-II/I ratio is altered, indicating that CEE may be associated with abnormal autophagy. The GLP-1R agonist semaglutide in turn reverses the expression of these proteins. Here, we suggest that the GLP-1R agonist Semaglutide reverses the downregulation of Parkin and PINK1 induced by CEE, which may contribute to the restoration of normal mitophagy activity. Furthermore, we suggest that GLP-1R agonist Semaglutide may enhance mitophagy by upregulating LC3B while downregulating P62. Several limitations should be noted regarding the assessment of autophagy and mitophagy. First, our conclusions are based on Western blot analysis of LC3-II/I ratio and P62 expression, which are indirect indicators of autophagic activity. Static measurements of these markers cannot distinguish between enhanced autophagosome formation and impaired lysosomal degradation without the use of lysosomal inhibitors (Gottlieb et al., 2015). Second, while changes in Pink1 and Parkin expression suggest alterations in the mitophagy pathway, direct visualization of mitochondria within autophagosomes by TEM would provide more definitive evidence of mitophagy flux (Kaludercic et al., 2020). Due to sample limitations, we were unable to perform such TEM analysis in the current study. Future studies using TEM-based quantification are warranted to confirm the effects of semaglutide on mitophagy flux.

In psychiatric disorders such as anxiety, neuroplasticity is crucial, with synaptic plasticity being a key component of this process (Gosselink et al., 2022; Ren et al., 2025). Simultaneously, maintaining mitochondrial health and efficient function is crucial for neuronal integrity, viability, and synaptic activity (Cardanho-Ramos and Morais, 2021). Alcohol can lead to synaptic abnormalities, thereby contributing to various neuropsychiatric disorders including anxiety (Roberto and Varodayan, 2017; Socodato et al., 2020). In our study, we found that the GLP-1R agonist semaglutide restored the CEE-induced downregulation of GLP-1R expression in prefrontal cortical neurons. Moreover, we found that GLP-1R can regulate mitochondrial function. Therefore, we further investigated the effect of GLP-1R on neuronal synaptic function. We observed that Semaglutide administration in the PFC ameliorated the CEE-induced downregulation of the synaptic proteins PSD95 and SYN, also restored structural damage in dendritic architecture. These results indicate that the GLP-1R agonist Semaglutide ameliorates CEE-induced neuronal synaptic impairment, thereby alleviating anxiety-like behaviors in CEE mice.

The limitation of this study is that we did not include a semaglutide-only group without ethanol exposure. As our primary objective was to evaluate the therapeutic potential of semaglutide in reversing CEE-induced anxiety and mitochondrial dysfunction, the appropriate comparison was between the disease model (CEE) and the treated disease model (CEE + Sema), with water-fed animals serving as baseline controls. Moreover, previous studies have demonstrated that semaglutide does not alter anxiety-related behaviors or locomotor activity in healthy rodents (Zhang et al., 2025; Boboc et al., 2024), consistent with the absence of pathological phenotypes in naïve animals. Thus, while the inclusion of a semaglutide-only group would have allowed assessment of potential intrinsic effects, it was not essential for addressing our primary research question. Future studies may incorporate such a group to further characterize the baseline effects of GLP-1R activation. In this study, we used CEE paradigm followed by a withdrawal period. While this model is widely used to study withdrawal-induced anxiety, it does not allow us to dissociate the effects of ethanol exposure *per se* from those of withdrawal. Future studies incorporating a continuous ethanol exposure group without withdrawal would help determine whether the observed changes in GLP-1R expression and mitochondrial function are specifically attributable to withdrawal or to cumulative ethanol exposure. However, in this study, we did not use a selective GLP-1R antagonist (e.g., exendin-9–39) to confirm that the observed effects of semaglutide are specifically mediated by GLP-1R. While semaglutide is a well-characterized and highly selective GLP-1R agonist with minimal off-target activity (Le Roux and Mondoh, 2024; Melander et al., 2024), the inclusion of an antagonist would provide definitive evidence for receptor specificity. Future studies employing co-administration of semaglutide with a GLP-1R antagonist are warranted to further establish the causal role of GLP-1R signaling in the protective effects observed here.

In summary, our study suggests that enhancing the neuronal mitochondrial GLP-1R/CREB pathway may mitigate mitochondrial damage by regulating mitochondrial quality control, thereby attenuating anxiety-like behaviors in a CEE mouse model. Targeting GLP-1R activation may represent a promising therapeutic strategy for treating withdrawal-related anxiety in AUD.

## 5 Conclusion

Activation of GLP-1R alleviates anxiety-like behaviors in CEE mice. The GLP-1R/CREB pathway ameliorates mitochondrial functional impairment by modulating mitochondrial quality control in PFC neurons of CEE mice. Furthermore, GLP-1R activation mitigates neuron function in PFC induced by CEE. Collectively, these results suggest that activating the GLP-1R/CREB signaling pathway may represent a promising therapeutic strategy for preventing and treating alcohol withdrawal-induced anxiety-like behaviors.

## 6 Limitations

This study has several limitations. First, no semaglutide-only group or GLP-1R antagonist was used, leaving the specificity of the observed effects unconfirmed. Second, the ethanol withdrawal paradigm cannot distinguish between effects of ethanol exposure and withdrawal. Third, autophagy and mitophagy were assessed only by indirect markers (LC3-II/I ratio, P62, Pink1/Parkin) without lysosomal inhibitors or TEM, limiting conclusions about autophagic flux.

## Data Availability

The original contributions presented in the study are included in the article/Supplementary Material, further inquiries can be directed to the corresponding authors.
