# Supplementary material for: Activation of GLP-1R ameliorates alcohol withdrawal induced anxiety-like behavior by regulating neuronal mitochondrial quality control
Source: Front Pharmacol. 2026 May 29;17:1820128. doi: 10.3389/fphar.2026.1820128 (PMC13260387; doi:10.3389/fphar.2026.1820128)

Supplementary Material

# Materials and Methods

**Western blotting**

PFC dissection was performed on ice under a stereomicroscope. PFC tissues were isolated from brain tissues using RIPA lysis buffer (R0010; Solarbio). The protein concentration was measured using a bicinchoninic acid protein assay kit (ZJ102, Epizyme Biotech). A sample consisting of 12 μg of protein was loaded into each lane and separated by 8–12% SDS-PAGE gel. The separated proteins were transferred to PVDF membranes, which were blocked for 1 h at room temperature with 5% skim milk powder in TBST, and incubated with primary antibodies overnight at 4 °C. The following day, after four washes in TBST buffer for 5 min each, membranes were incubated with horseradish peroxidase (HRP)-conjugated goat anti-rabbit IgG (ZB-2305, 1:5000, Zhongshan Golden Bridge) and HRP-conjugated goat anti-mouse IgG (ZB-2301, 1:5000, Zhongshan Golden Bridge) for 2 h at room temperature. Following three washes with TBST buffer, the proteins were visualized using AI-800 System (GE, USA). The optical density of each band was then quantified using ImageJ software.

**Immunofluorescence**

Mice were deeply anesthetized with isoflurane and perfused with pre-cooled normal saline followed by 4% paraformaldehyde (PFA). Brains were extracted, fixed in 4% PFA for 24 h, and sequentially dehydrated in 20% sucrose for 24 h and 30% sucrose for another 24 h at 4°C. Brain tissues were sectioned at 40 μm slices using a freezing microtome and stored in cryoprotective solution at -20°C.

Slices were washed three times with PBS (5 min each). Slices were incubated in PBS containing 5% bovine serum albumin and 0.3% TritonX-100 for 2 h at room temperature. Slices were then incubated overnight at 4°C with primary antibodies against GLP-1R (GB113881, 1:5000, Servicebio), NeuN (GB11138, 1:5000, Servicebio). The slices were placed in PBS (PH7.4) (G0002, Servicebio) and washed on the pendulum table (SYC-Z100) for 3 times, 5 min each time. The corresponding HRP labeled (GB23302, 1:200, Servicebio; GB23303, 1:200, Servicebio) secondary antibody was added and incubated at room temperature for 50 min. The slices is placed in PBS (PH7.4) (G0002, Servicebio) and washed 3 times on the pendulum table (SYC-Z100) for 5 min each time. Drop the corresponding type of TSA (iF546-Tyramide G1251; iF488-Tyramide G1231; iF594-Tyramide G1242, Servicebio) and incubate at room temperature for 10 min. After incubation, the slices were placed in TBST (G0004) and washed by shaking on the decolorizing table for 3 times, 5 min each time. After slightly shaking off the excess liquid from the sections, add the immunostaining antibody stripping buffer (G1266,Servicebio) dropwise to completely cover the entire tissue. Incubate at room temperature for 5 min, and then remove the antibody stripping buffer. Add an adequate amount of the immunostaining antibody stripping buffer again to completely cover the tissue, and incubate at 37°C for 30 min. After the incubation is completed, place the slicess in TBST (G0004, Servicebio) and shake them on the pendulum shaker (SYC-Z100) for washing three times, 5 min each time. The above procedure was repeated three times. Following the antibody stripping steps, sections were incubated with DAPI (1:1000, Servicebio) in PBS for 10 min at room temperature. Sections were then washed three times with PBS (5 min each) and mounted with Prolong gold anti-fade reagent (G1401, Servicebio) and dried in the dark for 24 h. Slices were stored at -20°C until imaging. Images were acquired using a confocal microscope (Leica) and analyzed using ImageJ for quantification.

# Supplementary Figure

**Figure S1**


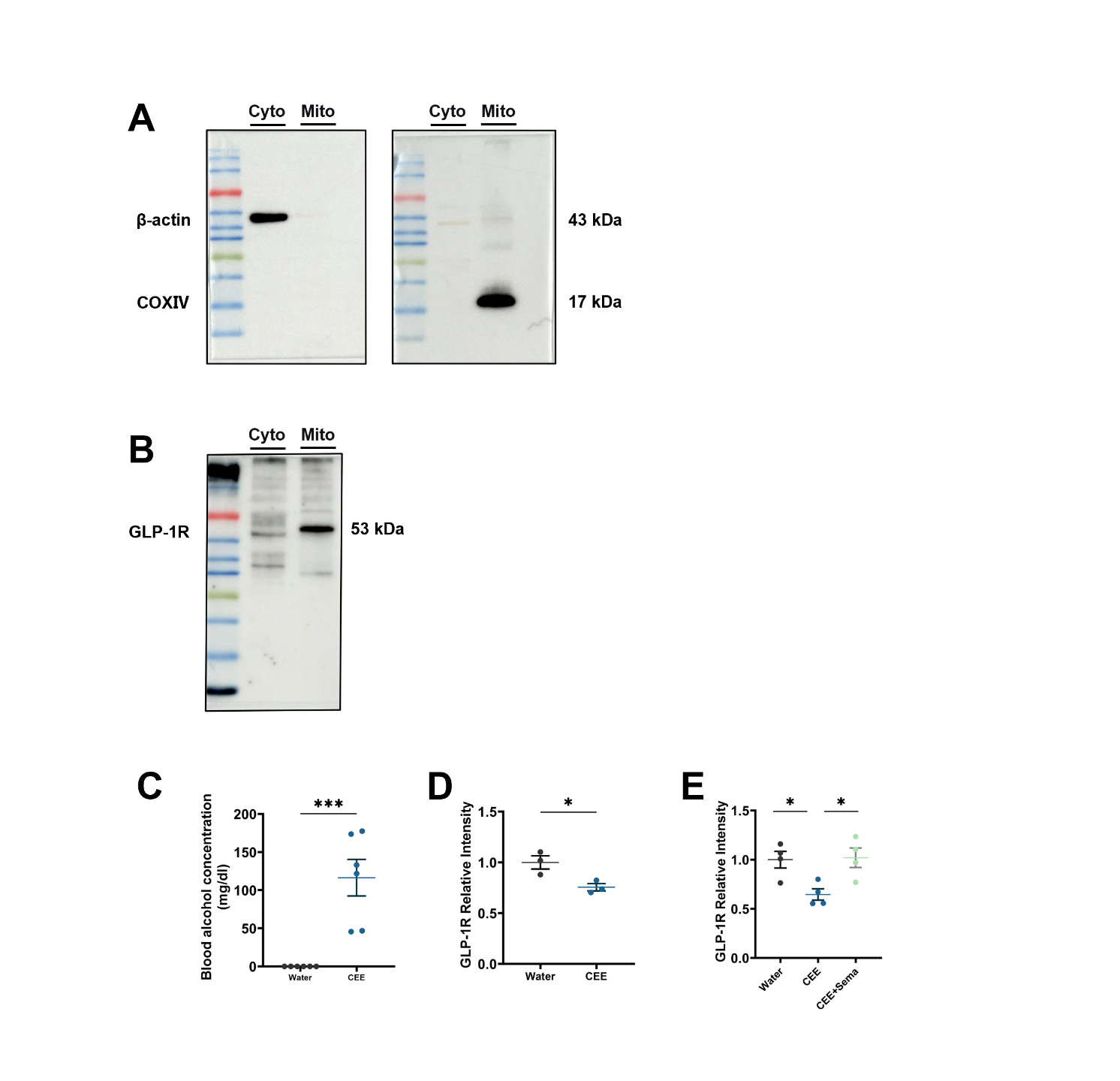
**Fig. S1.** (**A**) Protein expression of β-actin and COXIV were detected in both cytosolic and mitochondria on the same membrane by Western blot analysis. (**B**) Protein expression of GLP-1R were detected in both cytosolic and mitochondria by Western blot analysis. (**C**) Blood alcohol concentration after sacrifice (Two-tailed unpaired t test: t_(10)_ = 4.867, ***p = 0.0007. n = 6 mice per group). Data are presented as mean ± SEM. *p < 0.05, **p < 0.01, ***p < 0.001.

**Figure S2**


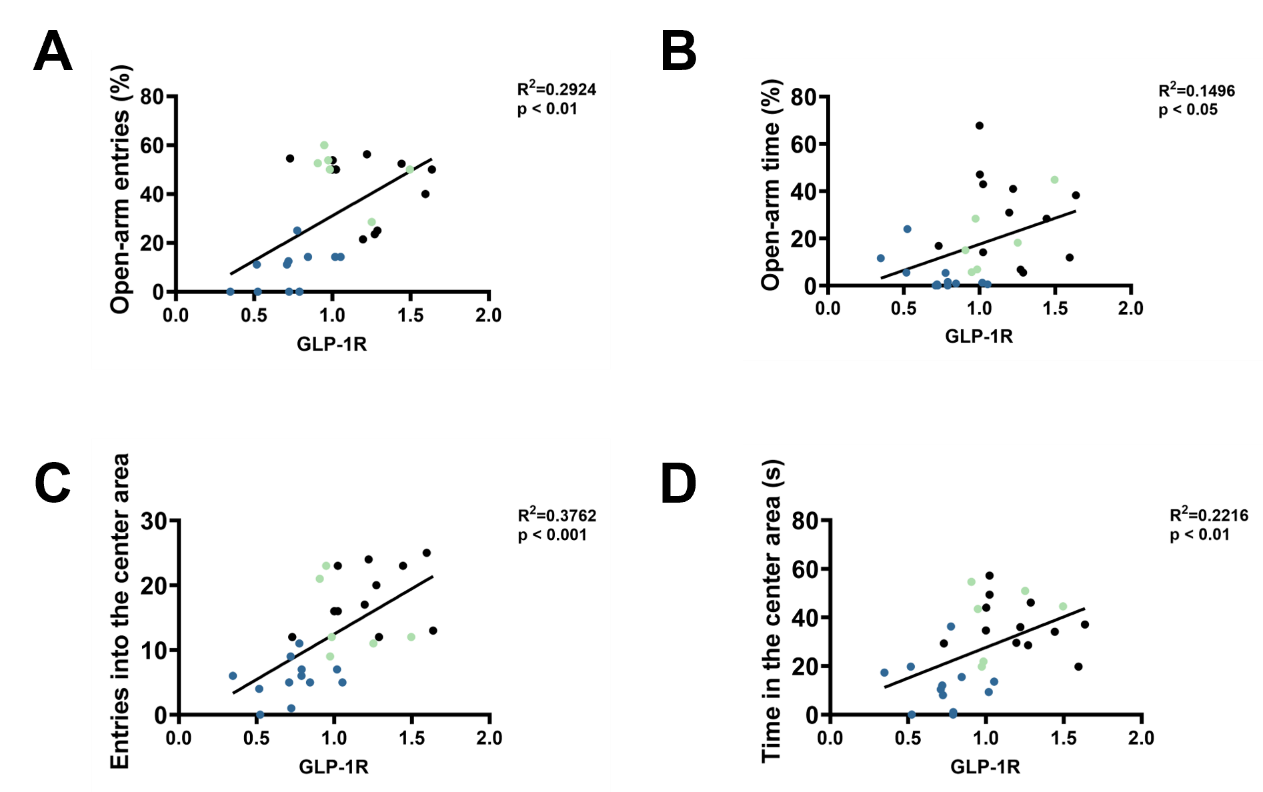

Supplement: Supplementary file 1 [file Supplementaryfile1.docx]
